# Tracheobronchial Tuberculosis Without Lung Involvement

**DOI:** 10.14740/jocmr2182w

**Published:** 2015-06-09

**Authors:** Jeronimo Campos, Glenda Ernst, Eduardo Borsini, Artemio Garcia, Miguel Blasco, Martin Bosio, Alejandro Salvado

**Affiliations:** a British Hospital, Respiratory Medicine Unit, Argentina

**Keywords:** Tracheobronchial tuberculosis, EBUS-TBNA

## Abstract

Endotracheal tuberculosis (ETTB) is an infrequent form of tuberculosis whose major feature is the infection of the tracheobronchial tree by *Mycobacterium tuberculosis*. This case presents a 73-year-old man admitted to our hospital with fatigue, weakness, dry cough and weight loss. His chest X-ray was normal but the high resolution computed tomography (HRCT) showed normal parenchyma images with mediastinal and hilar lymphadenopathy. There was inflammation of the tracheal wall and infiltrates in pavement epithelium; however, the tracheal biopsy for acid-fast bacilli was negative. He was finally diagnosed by endobronchial ultrasound-guided transbronchial needle aspiration (EBUS-TBNA) of the lymph nodes. Four drugs were prescribed and symptoms improved. EBUS-TBNA contributed to prompt diagnosis. The patient was treated and evolved without complications, such as tracheal stenosis.

## Introduction

Endotracheal tuberculosis (ETTB) is defined as granulomatous infection of the tracheobronchial tree with involvement of the bronchial mucosa and submucosa [1]. The normal diagnosis way from patients with active tuberculosis (TB) usually does not require a bronchoscopy as routine procedure. However, Jung and his colleagues recently described that ETTB is present in 50% of patients with active pulmonary TB. They performed the bronchoscopy in 429 TB patients finding 233 endobronchial tuberculosis (EBTB) [2]. EBTB often injures the tracheobronchial wall and leads to tracheobronchial stenosis [3].

The clinical presentation shows non-specific respiratory symptoms or radiologic images. Moreover, 10-20% of cases present with normal chest radiographs [4]. The diagnostic performance of the sputum smear microscopy is usually low and variable [5]; high resolution computed tomography (HRCT) works as a more sensitive tool and shows a classical pattern known as “tree-in-bud”. Fiber-optic bronchoscopy contributes to the evaluation and diagnosis of endotracheal obstructive lesions and the taking of the samples (biopsy or BAL) to the pathologic and bacteriological analyses [6]. However, diagnosis of ETTB remains challenging for clinicians since initially it shows non-specific signs and symptoms.

## Case Report

A 73-year-old man was admitted to the hospital with fatigue, weakness, dry cough and weight loss (around 6 kg) during the last 3 months, without fever or night sweating. He had past medical history of ex-smoker (35 packs/year), hypertension and hypothyroidism. He presented a myelodysplastic syndrome of 3-year progression. When he was 60 years old, he underwent resection of a melanoma in the lower right lobe.

Laboratory evaluation showed low number of leukocytes (1,900/mm^3^), a hematocrit of 31% and platelet count of 280,000/mm^3^. LDH was 414 U/L, erythrocyte sedimentation rate (ESR) was 100 mm and CRP was 20 mg/dL. The rest of the laboratory data were normal.

**Figure 1:**
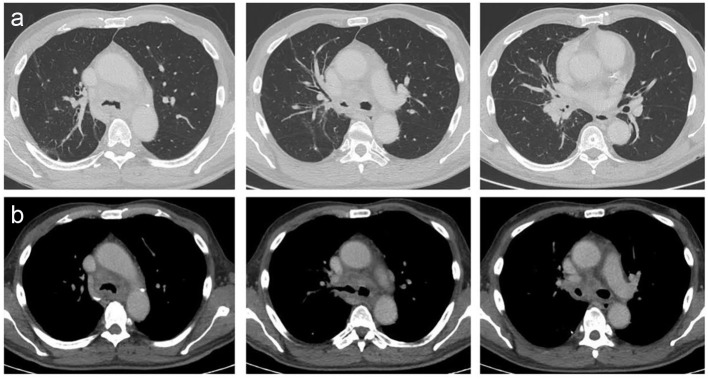
Tomographic images. (a) Normal lung parenchyma observed in different cuts of the HRCT. Commitment in the tracheal lumen with a narrowness in the right bronchus source due to an hilar mass. (b) Mediastinal windows observed in the HRCT. Tracheal bulge and adenomegalies in the mediastinum and hilum were observed.

**Figure 2:**
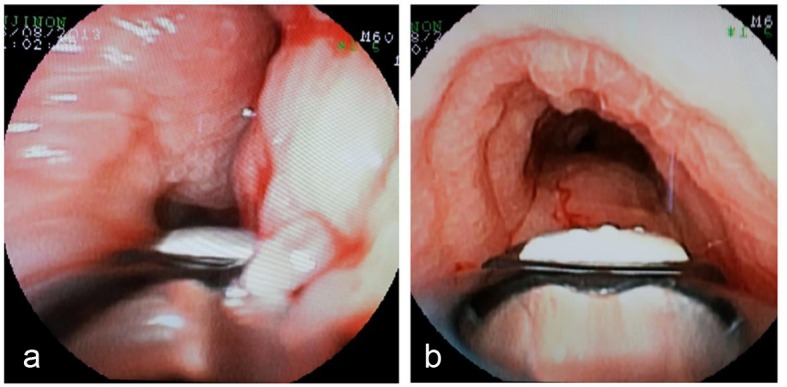
Bronchial images. (a) Bronchial mucosa edematous. (b) Infiltrated edematous and pavement of bronchial mucosa in the right bronchus source.

HRCT showed mediastinal and hilar lymphadenopathy with tracheal and right lobe involvement but no lung parenchymal involvement (Fig. 1). Fibrobronchoscopy showed the lower part of the tracheal wall with edema and infiltrates in pavement epithelium; however, tracheal biopsy for acid-fast bacilli and malignant cells was negative (Fig. 2). Symptoms worsened and endobronchial ultrasound-guided transbronchial needle aspiration (EBUS-TBNA) was performed to get samples of lymph nodes. The presence of necrotizing caseous granuloma confirmed the diagnosis of EBTB.

Patient was treated with isoniazid (5 mg/kg), rifampicin (10 mg/kg), pyrazinamide (25 mg/kg) and ethambutol (20 mg/kg) orally, during 9 months. Symptoms and serum inflammatory markers improved considerably.

## Discussion

The pathogenesis of EBTB remains poorly understood due to the heterogeneity in the clinical presentation, duration of symptoms and variability of clinical features depending upon the site, extent of involvement, and stage of the disease [3]. Systemic symptoms like anorexia, weight loss, and night sweats might not be prominent in EBTB [7].

Bronchoscopic findings contribute to classify this disease into several subtypes according to Chung classification. It has been previously reported that the therapeutic outcomes of each subtype can be predicted by follow-up bronchoscopy during the initial treatment [8].

Patients with suspicion of EBTB must be diagnosed promptly because they need immediate treatment to avoid development of tracheobronchial stenosis. The goal of treatment is eradication of tubercle bacilli. Corticosteroid therapy for prevention of bronchial stenosis in these patients remains controversial [9-11]. As stenosis from fibrous disease cannot be reversed with steroids or other medication, the only way to regain airway patency is through surgery or endobronchial intervention [12].

EBUS-TBNA is an invasive procedure that allows mediastinal and hilar lymph node assessment with high sensitivity. Currently, its role in the diagnosis of intrathoracic TB is being studied. Sun et al have recently described high sensitivity and specificity of EBUS-TBNA (85% and 100% respectively) in the diagnosis of EBTB [13, 14].

This case presentation reports a patient with a normal chest x-ray whose bronchoscopy showed granular EBTB. The endoscopic images presented granulomas in the tracheobronchial tree; however, the tracheal biopsy was negative. EBUS-TBNA of the lymph nodes is not considered a front-line diagnostic procedure for these cases; however, we present this case with a non-traditional diagnosis way. We decide to use alternatives tool due to EBTB suspicion. In this case, EBUS-TBNA contributed to confirm the presence of tubercle bacilli allowing for prompt treatment and avoiding complications such as tracheobronchial stenosis.
